# Advantages of FVIII-Extended Half-Life (Turoctocog Alfa Pegol) in the Management of Cardiac Surgery in a Patient with Mild Hemophilia A: A Case Report and Literature Review

**DOI:** 10.3390/hematolrep17040041

**Published:** 2025-08-06

**Authors:** Angela Napolitano, Andrea Venturini, Mauro Ronzoni, Graziella Saggiorato, Paolo Simioni, Ezio Zanon

**Affiliations:** 1 First Chair of Internal Medicine, Department of Medicine (DIMED), University of Padua, 35121 Padua, Italy; angela.napolitano@studenti.unipd.it (A.N.); graziella.saggiorato@unipd.it (G.S.); paolo.simioni@unipd.it (P.S.); 2 Cardiac Surgery Unit, Spedali Civili Hospital, University of Brescia, 25123 Brescia, Italy; andreaventurini67@gmail.com (A.V.); m.ronzoni001@unibs.it (M.R.)

**Keywords:** Cardiac surgery, hemophilia, FVIII-EHL

## Abstract

**Background and Clinical Significance:** Hemophilia A presents a considerable challenge in cardiac surgery due to the elevated risk of perioperative bleeding, particularly during procedures involving cardiopulmonary bypass. Standard management typically involves standard half-life (SHL) factor VIII (FVIII) concentrates, which require frequent dosing. Extended half-life (EHL) FVIII products offer theoretical advantages, including prolonged action and reduced infusion frequency, but their use in cardiac surgery remains largely undocumented. **Case Presentation:** We report the case of a 73-year-old male with mild Hemophilia A who underwent successful aortic valve replacement using a 25 mm Carpentier-Edwards Magna Ease biological prosthesis. The patient was managed perioperatively with an anti-hemorrhagic protocol based on EHL recombinant FVIII. The surgery and postoperative course were uneventful, with no bleeding complications or need for transfusion. **Conclusions:** This case illustrates the potential role of EHL FVIII in safely managing hemophilic patients undergoing major cardiac surgery. Given the lack of existing reports in the literature, further studies are warranted to evaluate the efficacy and safety of EHL FVIII in this setting and to potentially optimize perioperative care protocols for this patient population.

## 1. Background and Clinical Significance

Hemophilia A is a congenital bleeding disorder characterized by a deficiency of clotting factor VIII, which significantly increases the risk of hemorrhage during surgical procedures. Cardiac surgery, especially when involving cardiopulmonary bypass, presents unique challenges in these patients due to the complexity of balancing adequate anticoagulation and bleeding prevention. Traditionally, the perioperative management of hemophilia A patients has relied on standard half-life (SHL) factor VIII concentrates, necessitating frequent dosing and close monitoring to maintain hemostasis.

Recent developments in extended half-life (EHL) recombinant factor VIII products offer the potential for prolonged activity and reduced infusion frequency, which could simplify management and improve patient outcomes. However, there is a scarcity of documented cases describing the use of EHL factor VIII in cardiac surgery. This report describes the successful perioperative management of a 73-year-old patient with mild hemophilia A undergoing aortic valve replacement, utilizing an EHL factor VIII concentrate within a multidisciplinary care protocol. The case aims to contribute valuable clinical insights into the feasibility and safety of EHL factor VIII in complex surgical settings.

The patient provided written informed consent for the publication of this case report and any accompanying images. The study protocol, including the consent collection procedure, was conducted in accordance with the principles of the Declaration of Helsinki.

## 2. Case Presentation

The case concerns a 73-year-old male patient with mild hemophilia A, with a factor VIII activity level of approximately 10%. The diagnosis was made at a young age, following the onset of right-eye proptosis caused by a carotid-cavernous fistula in 1979. He is currently receiving on-demand treatment with turoctocog alfa pegol 2000 U (26.3 U/kg). Previously, he was followed at another Hemophilia Center, where he reported never having received intravenous factor VIII supplementation. His bleeding history includes recurrent episodes of epistaxis during childhood and post-traumatic hemarthroses in the left knee, which were treated with arthrocentesis. In 1959, he sustained a right-frontoparietal head trauma. In 1988, he underwent a hemorrhoidectomy that was complicated by bleeding on the third postoperative day, requiring transfusion of two units of fresh frozen plasma. He has also had inguinal hernia repair and arthroscopies of both the right and left knees. Dental procedures were successfully managed with tranexamic acid at a dose of 13.15 U/kg (500 mg, two tablets three times daily) without significant bleeding episodes. From a family history perspective, it is noted that a maternal uncle died because of post-traumatic bleeding.

The patients presented with fatigue and dizziness for several months, without chest pain, palpitations, syncope, or other anginal equivalents. Due to the persistence of symptoms, he visited his general practitioner, who, during the cardiac examination, detected a diastolic murmur (grade 3/6) at the aortic focus. An echocardiogram was performed, revealing fibrocalcific degeneration of the semilunar valves, severe regurgitation with a large posterior-directed jet, and severe aortic insufficiency. No abnormalities were noted on the ECG.

Preoperative evaluations included a coronary angiography, which revealed mild-to-moderate stenosis and a 50–70% narrowing of the left anterior descending (LAD) artery. Prior to the procedure, the patient was referred to our Hemophilia Center and, in accordance with our recommendations, received 2000 U (26.3 U/kg) of turoctocog alfa pegol in combination with tranexamic acid (13.15 U/kg IV). The procedure was carried out successfully and without any complications.

As part of the preoperative workup, a CT angiogram of the chest and abdomen was performed, revealing minimal evidence of aortic atherosclerosis. Additionally, a carotid artery ultrasound (ecoTSA) and global spirometry were conducted, both of which showed no significant abnormalities.

The case was discussed in a multidisciplinary Heart Team meeting, and the decision was made to correct the aortic valve dysfunction with the implantation of a 25 mm CE Magna Ease biological prosthesis via median sternotomy. An anti-hemorrhagic protocol was established with the use of turoctocog alfa pegol, a recombinant FVIII with an extended half-life.

The anti-hemorrhagic regimen comprised intravenous administration of tranexamic acid at a dose of 13.15 U/kg and factor VIII at 6000 IU (78.9 IU/kg) administered 30 min prior to the procedure. A subsequent dose of tranexamic acid (13.15 U/kg IV) and factor VIII (2000 IU IV, 26.3 IU/kg) was given 12 h post-procedure. On postoperative days 1 and 2, tranexamic acid (13.15 U/kg IV) and factor VIII (2000 IU IV, 26.3 IU/kg) were administered twice daily. From postoperative day 3 to day 7, oral tranexamic acid (13.15 mg/kg) was administered three times daily, alongside intravenous factor VIII (2000 IU, 26.3 IU/kg). Coagulation parameters, including prothrombin time (PT), activated partial thromboplastin time (aPTT), and factor VIII activity levels, were assessed daily throughout the treatment period.

On September 16, the patient underwent cardiac surgery for aortic valve replacement with the implantation of a 25 mm CE Magna Ease biological prosthesis via median sternotomy, under general anesthesia and with the assistance of cardiopulmonary bypass. The surgery and the subsequent period in intensive care were free of complications.

**Figure 1:**
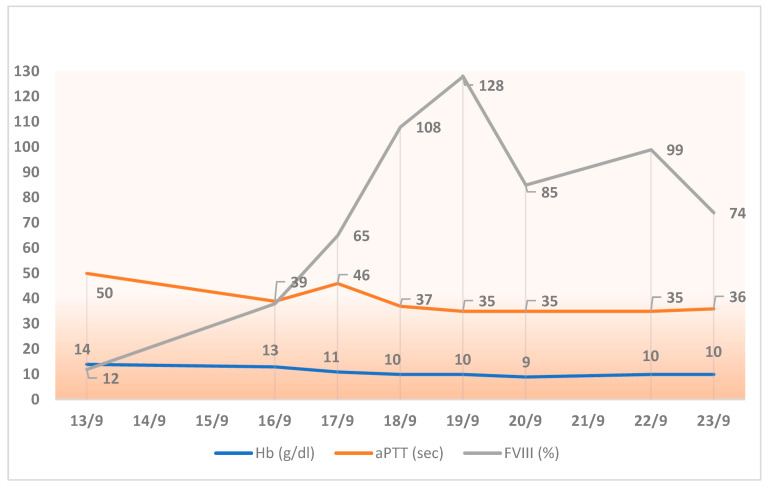
Trends in hemoglobin, aPTT, and FVIII activity during the week following cardiac surgery (performed in September 2016).

For the assessment of factor VIII activity, a chromogenic assay was used, which is the reference method for turoctocog alfa pegol. Baseline factor VIII levels were 12%, with an aPTT of 50 s, and on the day of surgery, the levels had increased to 65% with an aPTT of 46.7 s. Over the following 5 days, the average factor VIII levels were 98.8% with an aPTT of 36.14 s. Baseline Hb levels were 14 g/dL, dropping to 11 g/dL on the day of surgery and to 10 g/dL 4 days after the surgery (Figure 1).

The surgery and postoperative period in the intensive care unit were uneventful. No blood transfusions were necessary. The patient was extubated in the fourth postoperative hour and transferred to the ward on 17 September 2024. After the implantation of the biological aortic prosthesis, aspirin was prescribed for 3 months, with no complications.

From 23 September to 7 October, cardiac rehabilitation was carried out, during which the patient continued receiving FVIII 2000 IU (26.3 IU/kg) three times a week. After the implantation of the biological aortic prosthesis, aspirin was prescribed for 3 months, with no complications. At one year postoperatively, the patient remains free of any cardiac-surgery-related adverse events or late complications.

## 3. Discussion and Literature Review

Hemophilia A is classified based on the level of factor VIII (FVIII) activity: mild (5–40%), moderate (1–5%), and severe (<1%). Patients with severe hemophilia are at high risk for spontaneous bleeding and face significant hemorrhagic complications during surgical procedures. Even individuals with mild or moderate hemophilia may experience considerable bleeding challenges during major interventions such as cardiac surgery, where hemostatic demands are particularly high [1]. Cardiac surgery in hemophilia patients presents unique challenges due to the use of cardiopulmonary bypass (CPB), which results in hemodilution, platelet loss, and consumption of clotting factors, including FVIII. To mitigate perioperative bleeding risk, the World Federation of Hemophilia (WFH) recommends maintaining FVIII activity levels of 80–100% on the day of surgery, gradually tapering over the postoperative period. Replacement therapy individualized using the standard formula allows for precise management. The required dose is determined using the following formula: Units required = body weight [kg] × desired increase of Factor VIII [% or IU/dL] × 0.5. The typical half-life of FVIII is approximately 12 h, although this can vary depending on the specific product used [2]. Currently, four EHL-rFVIII products have been approved: three obtained by pegylation (damoctocog alfa pegol/BAY 94-9027—Jivi, rurioctocog alfa pegol/BAX 855—Adynovate, turoctocog alfa pegol/N8-GP—Esperoct) and one by Fc fusion (efmoroctocog alfa—Elocta/Eloctate). Extended half-life recombinant factor VIII concentrates (EHL-rFVIIIs) have been shown to offer a significantly longer half-life—approximately 1.4 to 1.6 times that of standard half-life (SHL) products. This pharmacokinetic advantage allows for extended dosing intervals, enabling a more convenient and manageable treatment regimen. By reducing injection frequency, EHL-rFVIII supports optimal prophylaxis, enhances treatment adherence, and improves patients’ quality of life, all without compromising safety or efficacy. The longer time between doses also has the potential to substantially reduce the overall treatment burden. During surgery, EHL-rFVIII supports higher and sustained factor levels, allowing for less frequent dosing, improved prophylaxis, and possibly reduced overall FVIII consumption. Finally, EHL formulations currently represent a more cost-effective alternative to standard half-life (SHL) products, thanks to fewer required administrations and lower costs, while maintaining comparable efficacy and safety.

Most bleeding events resolve with 1–2 infusions in >96% of cases [3,4,5]. In an observational study conducted on 149 patients with hemophilia A receiving prophylaxis with the extended half-life recombinant factor VIII (EHL-rFVIII) damoctocog alfa pegol for a minimum duration of six months, favorable clinical outcomes were observed. Overall, 75% of patients reported no bleeding events, while 86% reported a reduction in infusion frequency, and the average factor VIII consumption (IU/kg) decreased by approximately 17.5%, both on a monthly and annual basis [6].

These findings support the clinical efficacy and therapeutic efficiency of EHL-rFVIII products in real-world clinical practice.

Similarly, in our clinical case, the administration of turoctocog alfa pegol, an extended half-life recombinant FVIII (EHL-rFVIII), proved advantageous when compared with a standard FVIII regimen.

**Figure 2:**
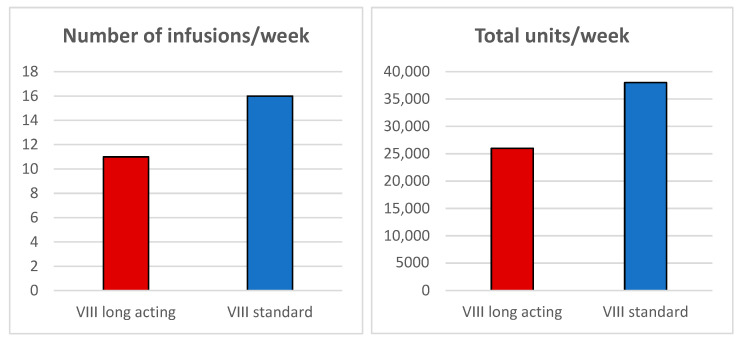
Comparison of the number of infusions/week and total units/week between extended half-life (EHL) FVIII and standard half-life (SHL) FVIII.

Administering a dose of 78.9 IU/kg allowed us to achieve a stable preoperative FVIII activity around 65%, thanks to a rigorously personalized treatment protocol. This individualized approach, supported by careful clinical and laboratory monitoring, ensured effective hemostatic control, significantly reducing the risk of bleeding and thrombotic events, while also limiting the overall consumption of FVIII compared with traditional products. The patient required only 11 infusions, compared to the 16 infusions typically needed with conventional FVIII, representing a 31.25% reduction in infusion frequency. Furthermore, the total number of international units (IUs) administered was significantly lower, amounting to 26,000 IU versus 38,000 IU, corresponding to a 31.5% reduction (Figure 2).

While EHL-rFVIII has proven effective in orthopedic and general surgeries [7,8], data in cardiac surgery remain sparse, largely limited to case reports and small series [9]. Given the specific challenges of CPB, including heparinization, hypothermia, and consumption of multiple clotting factors, tailored FVIII replacement strategies are crucial. With the significant improvement in life expectancy among patients with hemophilia—primarily due to advances in replacement therapy and multidisciplinary management—age-related comorbidities, particularly cardiovascular diseases, have become increasingly prevalent in this population. As a result, hemophilic patients are undergoing cardiac surgical procedures with growing frequency. These interventions commonly include coronary artery bypass grafting (CABG), aortic valve replacement, and mitral valve repair. Such procedures pose unique perioperative challenges due to the intrinsic bleeding risk associated with hemophilia, the need for precise factor VIII (FVIII) replacement strategies, and the potential requirement for postoperative antithrombotic therapy. When valve replacement is necessary, bioprosthetic valves are preferred for their lower thrombogenicity, minimizing the need for long-term anticoagulation [10,11,12]. In select cases, On-X mechanical valves may be used due to their reduced anticoagulation requirements [13]. Minimally invasive and transcatheter approaches, such as TAVR and robotic-assisted surgery, have demonstrated feasibility and safety in this population, offering reduced bleeding risk and faster recovery [14,15]. In our patient, considering age and comorbidities, particularly considering the bleeding history, a bioprosthetic aortic valve was selected. This choice was made to avoid long-term anticoagulation and mitigate bleeding risk, in accordance with current recommendations for valve replacement in patients with bleeding disorders.

Post-surgery, antithrombotic regimens must balance bleeding and thrombosis risks. VKA therapy requires FVIII ≥ 20%, while aspirin may be used with FVIII ≥ 1–5%. This necessitates close FVIII monitoring and multidisciplinary coordination between hematology and cardiology teams. In select high-risk cases, low-dose anticoagulation has been successfully administered alongside FVIII prophylaxis [10,16]. In the case of the patient in our clinical report, we decided to administer aspirin for 3 months, which proved to be effective as the patient did not experience any complications. This approach was chosen to balance the need for antithrombotic therapy while minimizing the risk of bleeding, considering the patient’s history and the type of valve replacement.

In patients with hemophilia A and in the presence of inhibitors, the use of bypassing agents such as activated prothrombin complex concentrate (APCC) and recombinant activated factor VII (rFVIIa) may represent a therapeutic option in cases of refractory bleeding, including those occurring after cardiopulmonary bypass (CPB) during cardiac surgery. However, the use of these agents requires caution, as it has been associated with an increased risk of thrombotic events in this clinical setting. Intensive FVIII exposure, such as during surgery, can trigger inhibitor formation, particularly in previously untreated patients or in those with severe hemophilia. Although rare, inhibitors can arise postoperatively, complicating future hemostatic management [17,18,19]. In our patient, the possibility of being a PUP (Previously Untreated Patient) must be considered; however, the inhibitor assay after surgical treatment was negative, consistent with the 0.03% immunogenicity rate reported for turoctocog alfa pegol.

The available literature on cardiac surgery in patients with hemophilia A revealed a significant limitation in the description of replacement therapy strategies, particularly regarding the specific factor VIII (FVIII) concentrates used. In many published case reports and series, the product type was not specified; when mentioned, it was almost exclusively standard half-life (SHL) concentrates (Table 1). This trend indicated an underutilization of newer extended half-life (EHL) products, despite their potential pharmacokinetic and clinical advantages. In the study by MacKinlay et al., which documented 12 cardiovascular procedures in hemophilic patients, no details were provided on the type of FVIII used. Notably, during aortic valve replacement in a patient with moderate hemophilia, significant bleeding resulted in hypotension and necessitated reoperation [20]. The absence of information on the replacement therapy used significantly hindered any meaningful comparison of FVIII product efficacy. De Bels et al. reported the use of 50 IU/kg of FVIII followed by continuous infusion in a patient with mild hemophilia. Although the specific product was not disclosed, the intensity of the regimen strongly suggested the use of an SHL concentrate [21]. A particularly complex case was described by Ghosh et al., involving a patient with inhibitors and hemorrhagic complications during mitral valve replacement. Once again, the FVIII type was not identified, but the severity of bleeding underscored the need for more-effective protocols—potentially involving EHL concentrates [22]. Stine et al. documented the use of continuous infusion (4 IU/kg/h) with additional boluses over 7 days following an initial dose of 50 IU/kg. While the specific FVIII product was not mentioned, the treatment approach was consistent with SHL use. In this setting, an EHL concentrate could theoretically have reduced both the dosing frequency and the monitoring complexity [23]. Tang et al. represented one of the few studies in which the type of SHL used was clearly specified: Advate, Refacto, and Kogenate—all first- or second-generation recombinant products. No patients received EHLs. The therapeutic demand in patients with mild hemophilia (*n* = 4) ranged from 42,500 to 94,500 IU over 11–15 days, underscoring the high infusion burden associated with SHL products [24]. Lison et al. reported the use of Haemate P, a plasma-derived SHL FVIII, administered by continuous infusion for over 40 h. The intensity of the regimen once again reflected the pharmacokinetic limitations of SHL concentrates [25]. Zatorska et al. described a patient with baseline FVIII activity of 20%, treated with 66.6 IU/kg preoperatively and 45.5 IU/kg intraoperatively, without real-time monitoring of plasma FVIII levels. Although the specific concentrate was not mentioned, the dosing approach was in line with SHL use [26]. In the study by Chamos et al., Helixate FS, a recombinant SHL FVIII, was administered as a 50 IU/kg bolus followed by continuous infusion over 7 days [14]. This case reinforced the need for complex regimens to maintain therapeutic FVIII levels with SHL products. Shalabi et al. presented a case series in which, with few exceptions, the specific FVIII products used were not reported. This lack of detail significantly limited clinical interpretation and the extraction of evidence-based recommendations [27]. Kang et al. described a patient successfully treated after gene therapy, with reduced infusion needs and sustained high FVIII levels [28]. This suggested a new therapeutic model, potentially analogous to the long-acting effect of EHL concentrates.

Bleeding risk in cardiac surgery for hemophilic patients depended on three main factors: the severity of hemophilia, the presence of FVIII inhibitors, and the need for postoperative antithrombotic therapy.

In patients with mild or moderate hemophilia A, bleeding risk was relatively low, particularly when FVIII levels were adequately maintained. Tang et al. [24], Gasparovic et al. [29], and MacKinlay et al. [20] reported successful aortic valve replacements (AVRs) ± CABG or even double valve surgeries without major bleeding in this group.

Conversely, in patients with severe hemophilia A—even in the absence of inhibitors—bleeding risk was significantly higher, especially in surgeries with high hemostatic demands. For example, Diplaris et al. described a Bentall procedure for acute aortic dissection in a patient with severe hemophilia A requiring surgical re-exploration for bleeding on postoperative day (POD) 1, followed by further sternal bleeding managed conservatively [30].

The presence of inhibitors, even at low titers, represented a major risk factor for postoperative bleeding. Damodar et al. reported a successful AVR in a patient with severe hemophilia A and low-titer inhibitors (2.8 BU), managed with FEIBA and no complications [31]. In contrast, Ghosh et al. [22,32] described a previously undiagnosed patient who developed inhibitors postoperatively (2.4 BU) and experienced massive bleeding (hemoperitoneum, hemothorax, and shock). In such cases, bypassing agents such as rFVIIa or FEIBA may be effective but significantly increase management complexity.

Bleeding risk also depended on the type of surgery and need for anticoagulant or antiplatelet therapy. Mitral valve procedures (e.g., resection or annuloplasty) appeared to carry a lower hemorrhagic risk, even in severe hemophilia A. Xu et al. [33], Bhave et al. [34], and Tran et al. [15] reported favorable outcomes, even in complex cases, including one with high-titer inhibitors (12.4 BU), managed without intraoperative bleeding but with subsequent pleural effusions.

In procedures involving the ascending aorta and aortic root, bioprosthetic valves were generally preferred in hemophilic patients to avoid lifelong anticoagulation. Yildirim et al. [35] and Diplaris et al. [30] both described Bentall procedures using bioprostheses with only minor complications.

In contrast, cases involving mechanical prostheses—as described by Kaminishi et al. [36] and Bhave et al. [34]—required warfarin therapy, which led to hematuria and required INR adjustments. These cases were managed effectively, either surgically or conservatively. Examples included cardiac tamponade resolved with reoperation [37] and sternal wound bleeding in severe hemophilia A requiring either surgical or conservative treatment [30,38].

Late bleeding (i.e., occurring >72 h postoperatively) was more common in patients with relevant comorbidities (e.g., liver cirrhosis, coagulopathy, infections) and was often associated with antithrombotic therapy—particularly in cases of overdose or supratherapeutic INR. Clinical examples included gastrointestinal bleeding due to duodenal ulcer 13 days after discharge [24] and severe hematuria 5 weeks postoperatively in a patient with an INR of 2.9 [34].

In the reported case, there was no significant hemoglobin drop compared with the non-hemophilic population, and no transfusions of packed red blood cells (PRBCs) were required during the perioperative period. This favorable outcome was achieved by maintaining FVIII levels consistently above 30%, with a peak of 128%.

**Table 1:** Perioperative management and bleeding complications in cardiac surgery patients with hemophilia A.

References	Patient	Cardiac Surgery Type	Perioperative Treatment	Bleeding and Other Complications
MacKinlay N et al. (2000) [20]	12 cardiac surgical procedures performed on one carrier of hemophilia B and six patients with hemophilia A (baseline factor VIII levels ranging from 2% to 25%)	Cardiac catheterization, coronary artery bypass surgery, percutaneous transluminal coronary angioplasty, valve replacement, and closure of an atrial septal defect followed by pericardial effusion drainage	No specific details were provided regarding the type of factor VIII	During aortic valve replacement in a patient with moderate hemophilia, significant bleeding occurred, leading to hypotension and requiring reoperation
Kaminishi Y et al. (2003) [36]	53-year-old patient with mild hemophilia A	Severe aortic insufficiency and ascending aortic dilatation, successfully underwent a modified Bentall procedure, with implantation of a 28 mm Hemashield Gold vascular graft and a 25 mm Carbomedics mechanical valve	Simple bolus infusions of factor VIII concentrate before and after cardiopulmonary bypass-CrossEightMTM 50 IU/kg	No
Ghosh K et al. (2004) [22]	27-year-old patient with previously undiagnosed mild hemophilia A with FVIII inhibitors (2.4 BU)	Mitral valve replacement for severe rheumatic stenosis	No specific details were provided regarding the type of factor VIII	Major intra- and postoperative bleeding (hemoperitoneum, pericardial hematoma, hemothorax, and shock)
De Bels D et al. (2004) [21]	53-year-old patient with mild hemophilia A	Mitral valve replacement with a mechanical prosthesis (Carbomedics) and coronary artery bypass grafting (CABG) for grade III mitral regurgitation and two-vessel coronary artery disease	A 50 IU/kg bolus of S/D-treated factor VIII concentrate was given 2 h pre-op (FVIIIc 129%), followed by continuous infusion	No
Stine KC et al. (2006) [23]	64-year-old patient with mild hemophilia A	Mitral valve repair with annuloplasty and CABG for significant mitral insufficiency and coronary artery stenosis	No specific details were provided regarding the type of factor VIII	No
Tang M et al. (2009) [24]	Six patients with hemophilia A, including 1 with severe hemophilia, 1 with moderate hemophilia, and 4 with mild hemophilia	Coronary artery bypass grafting (CABG), aortic valve replacement, and more complex procedures such as CABG combined with aortic valve replacement and ventricular resection with mitral valve reconstruction	SHL-rFVIII concentrates used: Advate (*n* = 4), Kongenate (*n* = 1), Refacto (*n* = 1)	Postoperative bleeding from a duodenal ulcer in a 60-year-old patient with mild hemophilia A
Mannucci P.M. et al. (2010) [39]	45-year-old patient with mild hemophilia A	Aortic valve replacement with a mechanical prosthesis	No specific details were provided regarding the type of factor VIII	No
Lison S et al. (2011) [25]	67-year-old patient with moderate hemophilia A	Aortic stenosis (AS) and mild aortic regurgitation (AR)	Haemate	No
Zatorska K et al. (2012) [26]	30-year-old patient with mild hemophilia A	Severe acute mitral regurgitation caused by infective endocarditis due to methicillin-sensitive *Staphylococcus aureus* (MSSA), with perforation of the posterior mitral leaflet and prolapse of the anterior leaflet. The procedure included triangular resection of the anterior leaflet, quadrangular resection of the posterior leaflet, and mitral valve annuloplasty with implantation of a 28 mm Edwards Physio ring.	No specific details were provided regarding the type of factor VIII	No
Diplaris KT et al. (2012) [30]	54-year-old patient with severe hemophilia A	Acute type A dissection and a bicuspid aortic valve; patient underwent a Bentall procedure with a composite graft and biological valve (Biovalsalva)	Advate (Baxter SA, Lessines, Belgium), SHL—standard half-life rFVIII	The postoperative course was complicated by re-exploration for bleeding on postoperative day 1 (POD 1) and bleeding from the sternotomy site on POD 6, which was managed conservatively
Fitzsimons MG et al. (2013) [37]	53-year-old patient with mild hemophilia A and IgA deficiency	Aortic valve replacement	No specific details were provided regarding the type of factor VIII	Postoperative course was complicated by cardiac tamponade occurring 6 h after surgery; the chest was left open for 4 days to ensure hemostasis. Discharged on postoperative day 27 for rehabilitation. At 1-year follow-up: good exercise tolerance, no cardiac symptoms, minimal swallowing difficulties.
Quader M et al. (2013) [40]	63-year-old patient with mild hemophilia A	LVAD implantation (HeartMate II), aortic valve replacement (23 mm Magna), and CABG, followed by heart transplantation after 156 days	No specific details were provided regarding the type of factor VIII	GI bleeding, suspected pump thrombosis, and TIAs
Damodar S et al. (2014) [31]	23-year-old patient with severe hemophilia A, low-titer inhibitor	Rheumatic aortic stenosis patient who underwent aortic valve replacement (AVR)	No specific details were provided regarding the type of factor VIII	No
Merron B et al. (2015) [41]	84-year-old patient with mild hemophilia A	Severe aortic stenosis (AS) with transcatheter aortic valve replacement (TAVR) via the transfemoral approach	No specific details were provided regarding the type of factor VIII	No
Bhave P et al. (2015) [34]	13 of the 17 patients included had hemophilia A, with baseline FVIII levels ranging from 0 to 0.28 IU/mL	Coronary artery bypass grafting (CABG), aortic valve replacements, mitral valve repairs, aortic root replacements, and combined aortic valve replacement with CABG	FVIII replacement therapy with products such as Biostate or recombinant FVIII (Advate, Baxter Healthcare, Westlake Village, CA, USA), Kogenate (Bayer Healthcare, Leverkusen, Germany), Recombinate (Baxter Healthcare), or Xyntha (Pfizer, Collegeville, PA, USA)	No
Yildirim F et al. (2016) [35]	43-year-old patient with severe hemophilia and Marfan syndrome	Grade 3–4 aortic regurgitation with dilation of the aortic root and ascending aorta; underwent Bentall procedure	Haemoctin-SDH, plasma-derived factor VIII concentrate (pdFVIII)	No
Chamos C et al. (2017) [14]	57-year-old patient with severe hemophilia A	Aortic insufficiency secondary to infective endocarditis caused by *Staphylococcus epidermidis*; aortic valve with replacement with a Perimount Magna Ease bioprosthetic valve	Helixate FS, SHL—standard half-life rFVIII	No
Xu et al., (2019) [33]	54-year-old patient with severe hemophilia A	Mitral valve repair was performed with triangular resection of the posterior leaflet, placement of an artificial chordae using GORE-TEX CV-4, and implantation of a No. 28 Sorin annuloplasty ring. The procedure was combined with coronary artery bypass grafting (CABG).	rFVIII, Bayer HealthCare LLC	No
Shalabi et al. (2020) [27]	Six patients had hemophilia A, including one with the severe form, one with moderate, and the remaining four with mild hemophilia	Coronary artery bypass grafting (CABG), and one patient also required a concomitant aortic valve replacement	No specific details were provided regarding the type of factor VIII	No
Cusano et al. (2022) [38]	54-year-old patient with severe hemophilia on emicizumab therapy	Infective endocarditis caused by *Streptococcus* group B, an aortic root abscess, and aortic insufficiency; patient underwent aortic valve replacement and aortic root repair	No specific details were provided regarding the type of factor VIII	Re-sternotomy was required due to bleeding from the sternal bone and muscles
Kang MY et al. (2022) [28]	60-year-old patient with severe hemophilia A who received gene therapy in October 2019	Coronary artery bypass grafting (CABG) with cardiopulmonary bypass (CPB)	Kovaltry, SHL—standard half-life rFVIII	No
Vander Zwaag S et al. (2024) [42]	80-year-old patient with severe hemophilia A (FVIII activity < 0.5%)	Coronary artery bypass graft (CABG) surgery with cardiopulmonary bypass (on-pump)	No specific details were provided regarding the type of factor VIII	No

## 4. Limits

A major limitation across the reviewed literature remained the lack of information on the type of FVIII concentrate used—whether plasma-derived, SHL recombinant, or EHL. This prevented the establishment of a clear relationship between replacement strategy and perioperative bleeding risk and hindered the evaluation of the comparative effectiveness of hemostatic protocols in the setting of cardiac surgery in patients with hemophilia A. Considering the publication dates of the included cases and the availability of extended half-life (EHL) factor VIII (FVIII) products on the market, it is reasonable to assume that most cases involved the use of standard half-life (SHL) FVIII concentrates, since EHL concentrates were only introduced starting in 2014. Nevertheless, analyzing these cases remains essential as they represent real-world clinical data and highlight the need for greater attention in the future to the precise identification of the concentrate type used. Another limitation concerns the small size of the analyzed cohorts: the available literature on cardiac surgery in patients with hemophilia mainly consists of small studies or case reports, limiting the generalizability of the results. Moreover, long-term follow-up data beyond five years on the use of EHL-FVIII in major surgeries are currently lacking. While acknowledging the need for studies with longer observation periods, we believe these initial clinical data are relevant in supporting the short- and mid-term safety of these therapies. Therefore, it is essential to promote further studies that address these limitations, paying particular attention to the type of concentrate used to enable a more detailed assessment of risks and benefits and including longer observation periods to confirm the long-term safety of these treatments.

## 5. Conclusions

The literature review indicates that perioperative management of hemophilic patients undergoing cardiac surgery mainly relies on standard half-life (SHL) FVIII concentrates, which require intensive regimens with high doses and frequent infusions to maintain adequate FVIII plasma levels.

This is the first documented case in the literature describing the use of an extended half-life factor VIII (EHL) in the treatment of a patient with hemophilia undergoing cardiac surgery.

Extended half-life (EHL) FVIII concentrates, though underutilized, offer several advantages, including prolonged action, fewer fluctuations in plasma levels, and reduced infusion and monitoring requirements. These benefits are particularly relevant in high-acuity settings like cardiac surgery.

Adopting EHL-based protocols could optimize perioperative care, but prospective, multicenter studies comparing SHL and EHL FVIII products are needed to provide robust evidence for clinical practice. Hemophilia poses a permanent bleeding risk, worsened during cardiac surgery due to cardiopulmonary bypass, heparin use, hypothermia, and hemodilution. Correcting coagulation defects is complex and depends on multiple factors, including hemophilia severity, inhibitors, and surgery type.

Protocols using EHL clotting factors should be prioritized over SHL products due to their extended duration of action, reducing administration frequency and enhancing safety by minimizing bleeding risks. Successful cardiac surgery in hemophilic patients requires personalized, multidisciplinary planning, with less invasive techniques and bioprosthetic valves preferred to reduce anticoagulation needs. When these are not possible, the On-X mechanical valve is a suitable alternative. Long-term monitoring by both cardiology and hematology specialists is essential for safe antithrombotic therapy management and optimal patient care.

## Data Availability

The original contributions presented in this study are included in the article. Further inquiries can be directed to the corresponding author(s).
